# Potential roles of 1,5-anhydro-d-fructose in modulating gut microbiome in mice

**DOI:** 10.1038/s41598-021-99052-y

**Published:** 2021-10-04

**Authors:** Takashi Ito, Takaaki Totoki, Seiya Takada, Shotaro Otsuka, Ikuro Maruyama

**Affiliations:** 1 Department of Systems Biology in Thromboregulation, Kagoshima University Graduate School of Medical and Dental Science, Kagoshima, Japan; 2 Department of Biomedical Laboratory Sciences, Faculty of Life Sciences, Kumamoto University, 4-24-1 Kuhonji, Kumamoto, 862-0976 Japan

**Keywords:** Microbiology, Health care

## Abstract

The gut microbiota has tremendous potential to affect the host’s health, in part by synthesizing vitamins and generating nutrients from food that is otherwise indigestible by the host. 1,5-Anhydro-d-fructose (1,5-AF) is a monosaccharide with a wide range of bioactive potentials, including anti-oxidant, anti-inflammatory, and anti-microbial effects. Based on its potential benefits and minimal toxicity, it is anticipated that 1,5-AF will be used as a dietary supplement to support general health. However, the effects of 1,5-AF on the gut microbiota are yet to be clarified. Here, using an unbiased metagenomic approach, we profiled the bacterial taxa and functional genes in the caecal microbiota of mice fed a diet containing either 2% 1,5-AF or a reference sweetener. Supplementation with 1,5-AF altered the composition of the gut microbiota, enriching the proportion of *Faecalibacterium prausnitzii*. 1,5-AF also altered the metabolomic profile of the gut microbiota, enriching genes associated with nicotinamide adenine dinucleotide biosynthesis. These findings support the potential benefits of 1,5-AF, but further studies are required to clarify the impact of 1,5-AF on health and disease.

## Introduction

The gut microbiota has tremendous potential to affect the host’s health. It plays an important role in educating the host immune system, providing protection against pathogen overgrowth, synthesizing vitamins, and generating nutrients from food that is otherwise indigestible by the host^1,2^. The diet and gut microbes are key determinants of the molecular composition of the host’s blood^3^, and dysbiosis of gut microbiota is linked to a wide range of chronic disorders, including metabolic syndrome and diabetes^4,5^.

For more than 200 million years, mammalian–microbial partnerships have adapted to local environments, in which climate, diet, and pathogens can all exert selection pressures^6^. It has become evident that environmental exposure is decisive in shaping the patterns of gut microbiota^7^. These patterns can be clustered into enterotypes, distinguished primarily by the levels of *Bacteroides* and *Prevotella*. The *Bacteroides* enterotype is associated with meat consumption, as in a Western diet, whereas the *Prevotella* enterotype is associated with a carbohydrate-based diet, as in agrarian societies or vegetarians^8^. However, the role of *Prevotella* in the host’s health is controversial. While some studies suggested an association of *Prevotella* with improved glucose metabolism^9,10^, others highlighted an association with insulin resistance and glucose intolerance^11^. Whether *Prevotella* is beneficial or deleterious may depend on the dietary environment or the diversity existing among different strains^12^. Although the causal relationships between the gut microbiota and age-associated disorders remain incompletely understood, recent studies have suggested that the strategic manipulation of the gut microbiota with prebiotics or probiotics can limit the progression of degenerative diseases in mice^13,14^.

1,5-Anhydro-d-fructose (1,5-AF) is a bioactive monosaccharide that is formed by the degradation of glycogen or related molecules. It occurs in mammalian tissues, algae, fungi, and bacteria, and is metabolized in vivo to 1,5-anhydro-d-glucitol (1,5-AG)^15^. 1,5-AG is the second most abundant polyol in human fluids, and can be used as a marker of glycaemic control in patients with diabetes. 1,5-AF has a wide range of bioactive properties, exerting anti-oxidant, anti-inflammatory, anti-microbial, anti-diabetic, and anti-cancer effects^15–18^. Based on its potential benefits and minimal toxicity^19^, it is anticipated that 1,5-AF will be used as a dietary supplement to support general health.

Given the anti-microbial, anti-diabetic, and anti-oxidant properties of 1,5-AF, it can be assumed that dietary supplementation with 1,5-AF will modulate the gut microbiota, with an indirect impact on the host’s health. However, to date, the influence of 1,5-AF on the gut microbiota remains unclear. Here, using an unbiased metagenomic approach, we profile the bacterial taxa and functional genes in the caecal contents of mice that consumed chow supplemented with 1,5-AF or a reference sweetener.

## Methods

### Study design

All experimental procedures complied with the ARRIVE guidelines and the Guideline for the Proper Conduct of Animal Experiments established by the Science Council of Japan, and were approved by the Institutional Animal Care and Use Committee of Kagoshima University, Kagoshima, Japan. Eight-week-old male C57BL/6J mice (CLEA Japan, Inc., Tokyo, Japan) were housed at room temperature (25–26 °C) under a 12-h light/dark cycle. The mice were allowed free access to water and a plant-polysaccharide-based chow (Oriental Yeast Co., Ltd, Tokyo, Japan) supplemented with either 2% 1,5-AF (SUNUS Co., Ltd, Kagoshima, Japan) or 2% erythritol as the reference supplement. The mice were randomly divided into the two groups each containing 8 animals: the 1,5-AF group and the control (erythritol) group. The sample size was estimated on the basis of preliminary unpublished data of the effects of dietary supplementation on the gut microbiota. The caecal luminal contents were harvested from the anesthetized mice at 10 weeks of age and stored in faecal collection tubes (TechnoSuruga Laboratory Co., Ltd, Shizuoka, Japan) until analysis.

### DNA extraction, library preparation, and sequencing

A shotgun metagenomic analysis was performed by Takara Bio Inc. (Kusatsu, Japan). The genomic DNA was extracted from the caecal luminal contents of the mice with a NucleoSpin Soil kit (Macherey–Nagel GmbH & Co., Düren, Germany). Shotgun libraries were prepared with a ThruPLEX DNA-Seq Kit (Takara Bio Inc.) and a DNA Unique Dual Index Kit (Takara Bio Inc.), according to the manufacturer’s protocols. These libraries were submitted for quality control with the Agilent 2100 BioAnalyzer (Agilent, Santa Clara, CA, USA) and 150-base pair paired-end sequencing using the Illumina NovaSeq 6000 platform (Illumina, Inc. San Diego, CA, USA).

### Metagenomic analysis

The metagenomic reads were pre-processed with KneadData v0.6.1, a computational tool designed to remove low-quality reads and contaminating host sequences. Approximately 7% of the 55,555,358 ± 8,347,087 raw reads were removed as low-quality reads, and then approximately 2% were removed as host reads, yielding 50,353,995 ± 7,683,757 putative microbiome reads. These reads were then taxonomically assigned using Kaiju v1.7.2 with NCBI nr database. The relative abundances of each bacterial taxon were calculated by dividing the total number of reads assigned to a particular taxon by the total number of reads for that sample. To determine variations in microbial communities between samples, principal coordinate analysis (PCoA) was performed based on Bray–Curtis distances using the QIIME software v1.8.0 and QIIME’s scripts (beta_diversity.py, principal_coordinates.py, make_emperor.py). Statistical significance of the differences between groups was calculated by PERMANOVA with 9,999 permutations using the QIIME script (compare_categories.py). Bacterial taxa that had differential abundance in the 1,5-AF group and the control group were determined by the Mann–Whitney test using the QIIME script (group_significance.py). To profile the gut microbial functions from the metagenomic sequencing data, the putative microbiome reads were mapped against the functional reference database UniRef90 using the Functional Mapping Analysis Pipeline (FMAP) v0.15 tool. Gene abundances were determined as reads per kilobase of exon per million mapped sequence reads (RPKM) and expressed as z-scores. The metagenomic analysis, including bioinformatic analysis, was performed by researchers in Takara Bio Inc. and Hokkaido System Science Co., Ltd., who were independent of the animal study.

### Statistical analyses

Mann–Whitney test or White’s non-parametric *t*-test^20^ was used to compare independent groups. For multiple comparisons, P values were adjusted with the false discovery rate (FDR) algorithm^21^. Corrected P values less than 0.05 were considered significant.

### Ethics approval and consent to participate

The experiments involving animals were approved by the Animal Care and Use Committee of Kagoshima University, Kagoshima, Japan.

## Results

### Dietary supplementation with 1,5-AF modulated the gut microbiota in mice.

**Figure 1:**
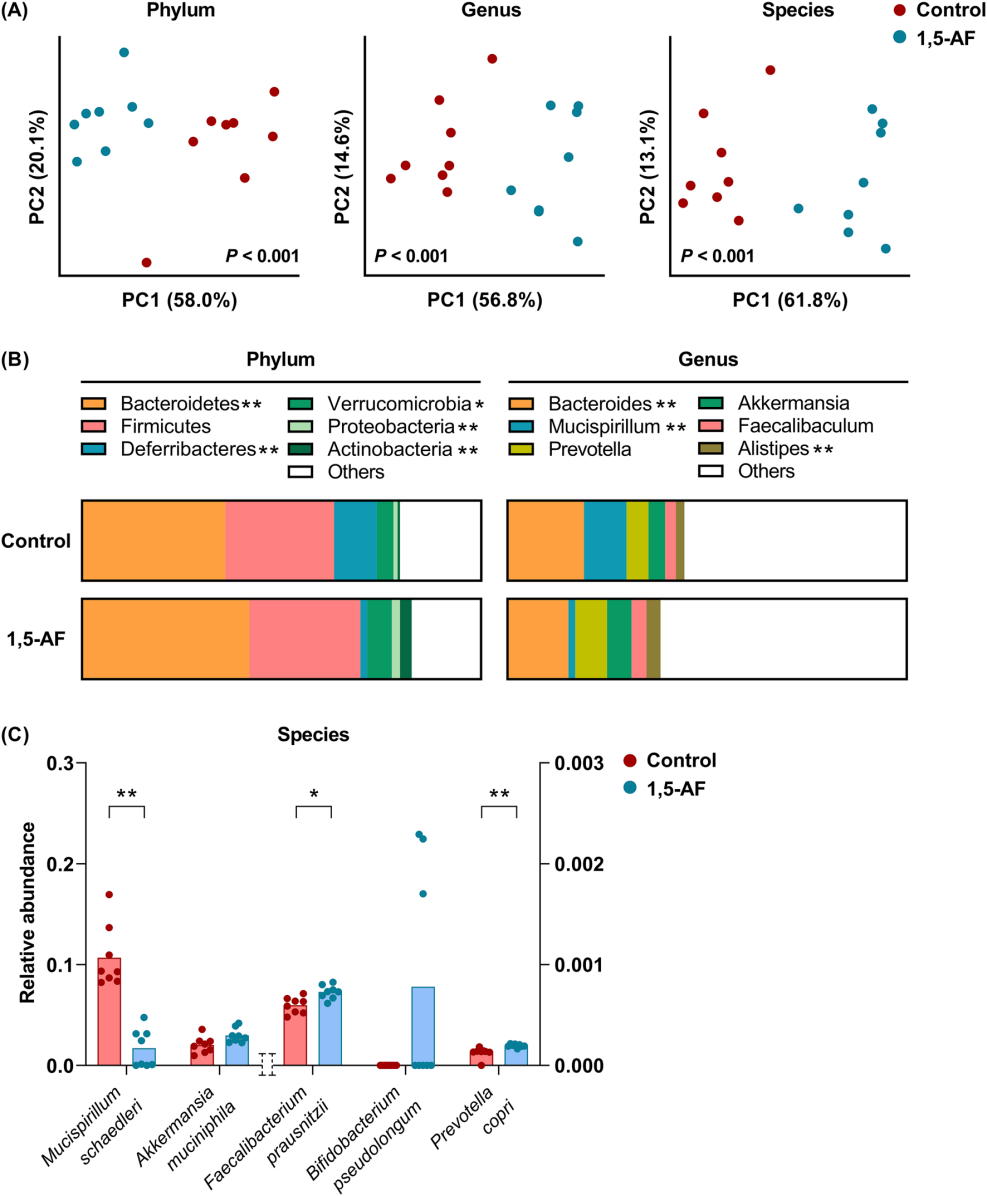
Dietary supplementation with 1,5-AF modulated gut microbiota in mice. (**A**) Principal coordinate analysis (PCoA) indicated that mice supplemented with or without 1,5-AF (n = 8, each group) were distinguishable by the structure of the gut microbiota at the phylum, genus, and species levels. Statistical significance of the differences between groups was calculated by PERMANOVA. (**B**) At the phylum level, relative abundance of Bacteroidetes was high in mice supplemented with 1,5-AF. Within Bacteroidetes, the genus *Bacteroides* was less abundant, whereas genera *Prevotella* and *Alistipes* were more abundant in mice supplemented with 1,5-AF. Mean values of the mice in each group (n = 8) are shown. (**C**) Relative abundances of each microbial species in mice supplemented with 1,5-AF (n = 8) were compared with those in control mice (n = 8). *M. schaedleri* and *A. muciniphila* are assigned to the left axis and *F. prausnitzii*, *B. pseudolongum*, and *P. copri* are assigned to the right axis. Bars indicate mean values. *P < 0.05 and **P < 0.01, determined by Mann–Whitney test with FDR adjustment for multiple comparison.

Among the 50,353,995 ± 7,683,757 putative microbiome reads detected in this study, 87% (43,824,256 ± 6,948,174 reads) were classified into 410 species, 124 genera, 54 families, 34 orders, 24 classes, 19 phyla, and three superkingdoms (Supplementary Material 1). The taxonomic variation across the samples was then analysed at the genus, phylum, and species levels. Principal coordinate analyses indicated that the structure of the gut microbiota in mice was distinguishable based on whether their chow was supplemented with 1,5-AF or not (Fig. 1A). This finding was consistent from the phylum level to the species level.

### The proportion of *Faecalibacterium prausnitzii* was higher in mice fed with 1,5-AF diet compare with those fed with control diet

Bacteroidetes and Firmicutes are the two major phyla in the gut microbiota. The proportion of Bacteroidetes was higher in mice fed with 1,5-AF diet than those fed with control diet (P < 0.01). The proportion of Firmicutes was similar in both groups (Fig. 1B). In the phylum Bacteroidetes, the proportion of genus *Bacteroides* was lower (P < 0.01), whereas the proportion of genus *Alistipes* was higher (P < 0.01) in mice fed with 1,5-AF diet compare with those fed with control diet. At the species level, the proportion of butyrate-producing bacteria *Faecalibacterium prausnitzii* was higher in mice supplemented with 1,5-AF (Fig. 1C), although that of *Roseburia* or *Butyrivibrio* was not. The proportion of *Bifidobacterium* was also high in some mice supplemented with 1,5-AF.

### Genes associated with nicotinamide adenine dinucleotide (NAD) biosynthesis were enriched in the gut microbiota of mice supplemented with 1,5-AF

**Figure 2:**
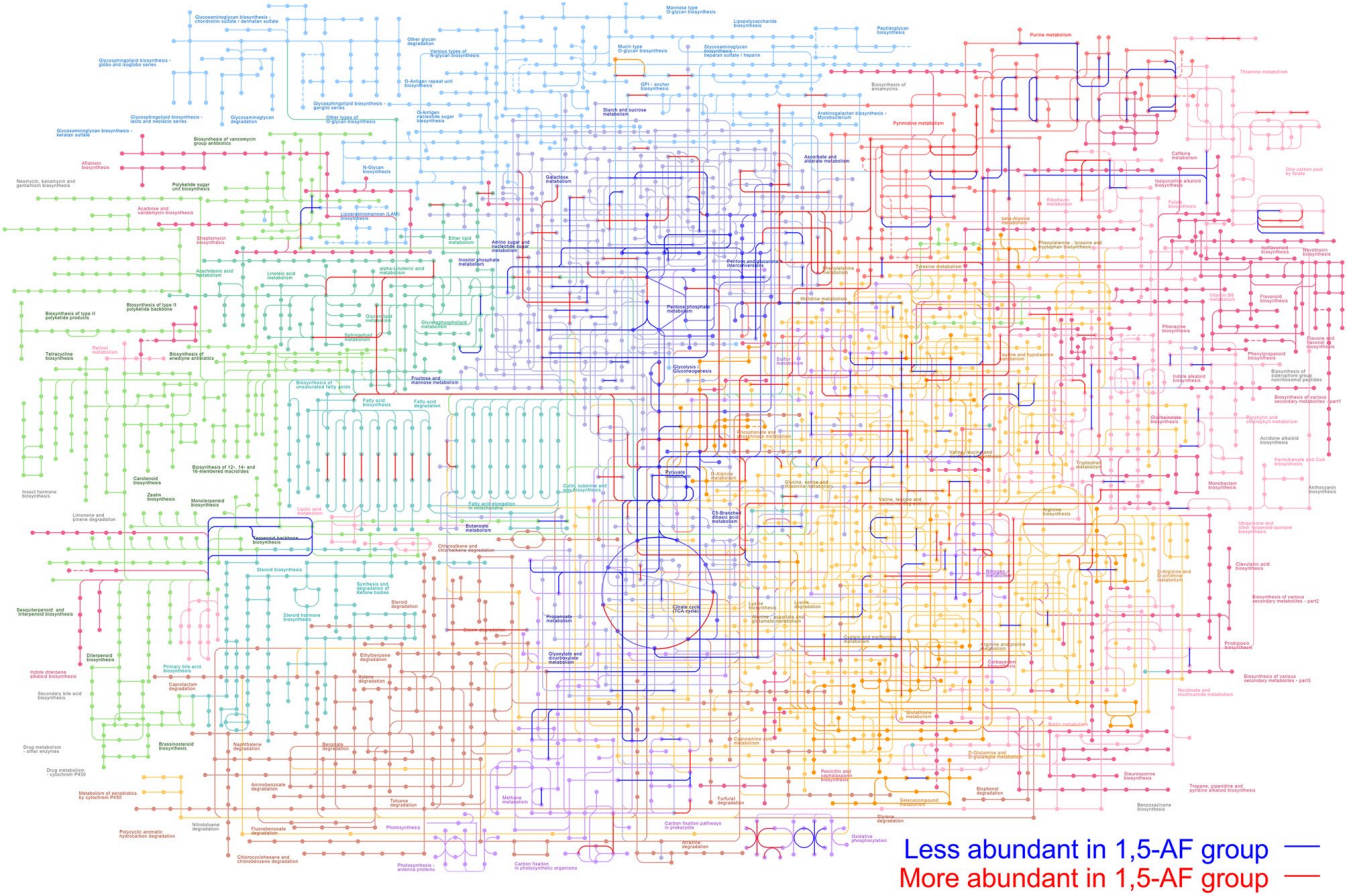
Comprehensive map of the metabolic pathways upregulated or downregulated by dietary supplementation with 1,5-AF. The metagenomic reads of the gut microbiota in mice treated with or without 1,5-AF (n = 8 in each group) were mapped against a functional reference database, and gene abundances were determined as reads per kilobase of exon per million mapped sequence reads (RPKM). The differences in RPKM values between the 1,5-AF-treated mice and the control mice were analysed using White’s non-parametric *t*-test with FDR adjustment for multiple comparison. Significantly more or less abundant genes in the gut microbiota of the 1,5-AF-treated mice are shown with red or blue lines, respectively. The metabolic map was created using the Kyoto Encyclopedia of Genes and Genomes (KEGG) mapper^28^ with the copyright permission.

**Figure 3:**
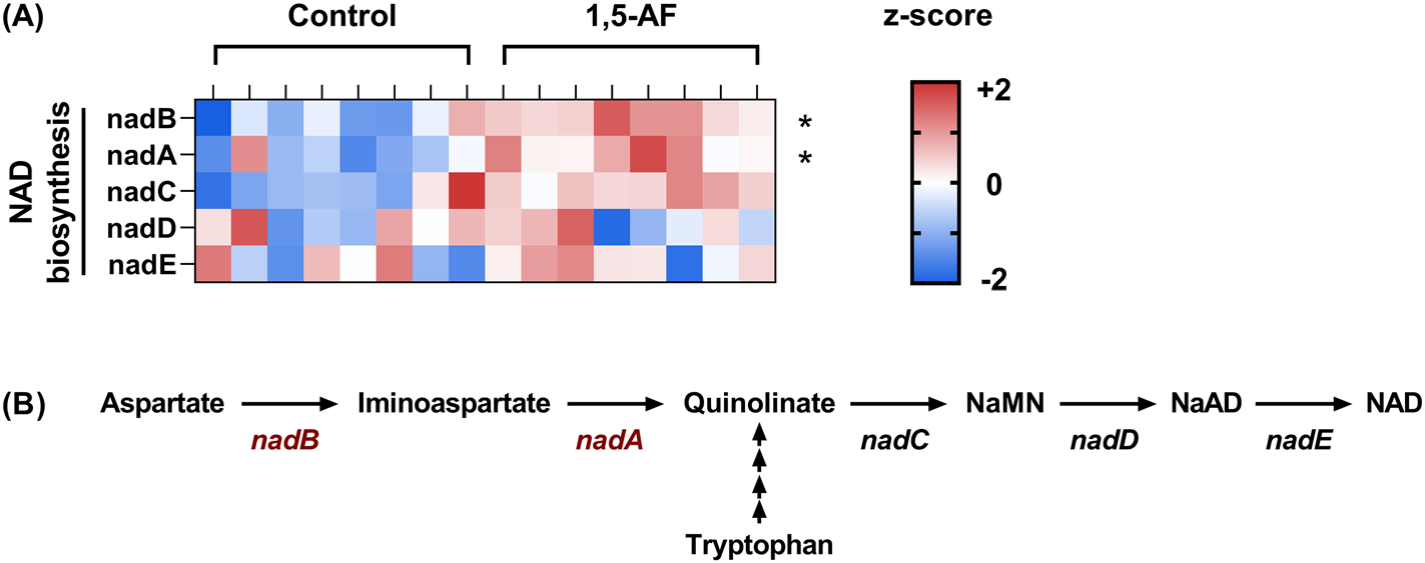
Genes associated with NAD biosynthesis were enriched in the gut microbiota of mice supplemented with 1,5-AF. (**A**) Relative gene abundances (z-scores) in NAD biosynthesis are shown; red and blue indicate high and low abundances, respectively. nad: nicotinamide adenine dinucleotide. *P < 0.05, determined by White’s non-parametric *t*-test with FDR adjustment for multiple comparison. (**B**) Schematic diagram of biosynthesis of NAD is shown. The genes encoding enzymes involved in the first two steps of the de novo synthesis of NAD from aspartate, were significantly enriched in the mice supplemented with 1,5-AF. NaMN: nicotinic acid mononucleotide. NaAD: nicotinic acid dinucleotide.

To profile the functional consequences of the gut microbial modification induced by 1,5-AF, the metagenomic sequencing data were mapped to the Kyoto Encyclopedia of Genes and Genomes (KEGG) Orthology database. In this analysis, 24,940,051 ± 3,866,382 reads were mapped to 3812 bacterial genes (Supplementary Material 2). Among these, 249 genes were significantly less abundant, and 232 genes were significantly more abundant in mice supplemented with 1,5-AF (Fig. 2). The more abundant genes included those associated with NAD biosynthesis (Fig. 3), such as *nadB* (P < 0.05) and *nadA* (P < 0.05).

## Discussion

In this study, we analysed the effects of 1,5-AF supplementation on the gut microbiota in healthy young mice. Supplementation with 1,5-AF altered (i) the composition of the gut microbiota, enriching *F. prausnitzii* and *Bifidobacterium*; and (ii) the metabolomic profile of the gut microbiota, enriching the genes associated NAD biosynthesis.

Nicotinamide and NAD are vitamins that participate in numerous redox reactions, including glycolysis, pyruvate-to-lactate and pyruvate-to-acetyl-CoA interconversions, β-oxidation, and oxidative phosphorylation. NAD also plays crucial roles in the regulation of metabolism and mitochondrial functions by acting as a co-substrate for NAD-dependent deacylases (sirtuins)^22^. NAD can be produced either through de novo synthesis or through salvage pathways from precursor molecules, including nicotinamide. In the de novo synthesis pathway, NAD is synthesized in sequential steps, starting from aspartate or tryptophan^23^. In the present study, the genes encoding nadB (l-aspartate oxidase) and nadA (quinolinate synthase), proteins involved in the first two enzymatic steps of the de novo synthesis of NAD from aspartate, were significantly increased in the mice supplemented with 1,5-AF (Fig. 3B). Because NAD can be synthesized from aspartate even in the absence of oxygen^23^, this pathway might be important in anaerobic NAD biosynthesis in the gut microbiota.

*F. prausnitzii* is a metabolically active commensal bacterium in healthy adults, and is considered a potential probiotic bacterium for human gastrointestinal diseases^24^. It is one of the most abundant butyrate-producing bacteria in the gastrointestinal tract, and by producing butyrate, *F. prausnitzii* may affect the intestinal-cell life cycle, pathogen resistance, and cancer progression. *Bifidobacterium* is considered a butyrogenic bacterium, and preferentially coexists with butyrate-producing bacteria such as *F. prausnitzii*^25^. During carbohydrate fermentation, *Bifidobacterium* produces acetate and lactate, which can be converted, in turn, to butyrate by *F. prausnitzii* in cross-feeding interactions. In this study, the relative abundance of both *F. prausnitzii* and *Bifidobacterium* was higher in mice fed with 1,5-AF diet compare with those fed with control diet, suggesting that 1,5-AF can be used to modulate the gut microbiota to counteract gastrointestinal diseases.

Our study had several limitations. First, it is unclear whether 1,5-AF directly stimulates the growth of specific bacteria associated with wellbeing or indirectly affects them through various host responses in the gastrointestinal tract^26^. Radioisotope tracing experiments showed that 63% of orally administered 1,5-AF was absorbed and renally excreted, 11% was found in the expired air, and 5% was found in the faeces^27^, suggesting that 1,5-AF may influence gut microbiota both directly and indirectly. Second, it is unclear whether the relative difference between the 1,5-AF group and the control (erythritol) group was due to the stimulatory effect of 1,5-AF or the inhibitory effect of erythritol. Third, dose–response effects of 1,5-AF were not examined in this study, and thus, the optimum dosage is uncertain. Fourth, it is unclear whether the effects of 1,5-AF are actually beneficial, neutral, or potentially harmful, because we used healthy young mice, which showed little change in their condition during the study period. In this context, we are planning to examine the effects of 1,5-AF on the gut microbiota of mice with age-associated diseases and inflammatory diseases. Further studies are required to clarify the potential effects of 1,5-AF in healthy and diseased organisms.

## Conclusions

Dietary supplementation with 1,5-AF modulated gut microbiota in mice. Genes associated with NAD biosynthesis were enriched in the gut microbiota of mice supplemented with 1,5-AF.

## Supplementary Information

Supplementary Information 1.

Supplementary Information 2.

## Data Availability

All data generated or analysed during this study are included in this published article and its Supplementary Information files.
